# Single-cell RNA sequencing reveals that lung mesenchymal progenitor cells in IPF exhibit pathological features early in their differentiation trajectory

**DOI:** 10.1038/s41598-020-66630-5

**Published:** 2020-07-07

**Authors:** Daniel J. Beisang, Karen Smith, Libang Yang, Alexey Benyumov, Adam Gilbertsen, Jeremy Herrera, Eric Lock, Emilian Racila, Colleen Forster, Brian J. Sandri, Craig A. Henke, Peter B. Bitterman

**Affiliations:** 1 University of Minnesota, Department of Pediatrics, Division of Pediatric Pulmonology, Minneapolis, USA; 2 University of Minnesota, Department of Medicine, Division of Pulmonary, Allergy, Critical Care and Sleep Medicine, Minneapolis, USA; 3 University of Manchester, School of Biological Sciences, Division of Cell Matrix Biology & Regenerative Medicine, Manchester, United Kingdom; 4 University of Minnesota, School of Public Health, Division of Biostatistics, Minneapolis, USA; 5 University of Minnesota, Department of Laboratory Medicine and Pathology, Minneapolis, USA; 6 University of Minnesota, Clinical and Translational Science Institute, Minneapolis, USA; 7 University of Minnesota, Department of Pediatrics, Division of Neonatology, Minneapolis, USA

**Keywords:** Mechanisms of disease, Computational biology and bioinformatics, Respiratory tract diseases

## Abstract

In Idiopathic Pulmonary Fibrosis (IPF), there is unrelenting scarring of the lung mediated by pathological mesenchymal progenitor cells (MPCs) that manifest autonomous fibrogenicity in xenograft models. To determine where along their differentiation trajectory IPF MPCs acquire fibrogenic properties, we analyzed the transcriptome of 335 MPCs isolated from the lungs of 3 control and 3 IPF patients at the single-cell level. Using transcriptional entropy as a metric for differentiated state, we found that the least differentiated IPF MPCs displayed the largest differences in their transcriptional profile compared to control MPCs. To validate entropy as a surrogate for differentiated state functionally, we identified increased CD44 as a characteristic of the most entropic IPF MPCs. Using FACS to stratify IPF MPCs based on CD44 expression, we determined that CD44^hi^ IPF MPCs manifested an increased capacity for anchorage-independent colony formation compared to CD44^lo^ IPF MPCs. To validate our analysis morphologically, we used two differentially expressed genes distinguishing IPF MPCs from control (CD44, cell surface; and MARCKS, intracellular). In IPF lung tissue, pathological MPCs resided in the highly cellular perimeter region of the fibroblastic focus. Our data support the concept that IPF fibroblasts acquire a cell-autonomous pathological phenotype early in their differentiation trajectory.

## Introduction

Idiopathic Pulmonary Fibrosis (IPF) is characterized by a multi-focal fibrotic reticulum that envelopes the alveolar gas exchange units resulting in death by asphyxiation^1^. The only approved therapeutic options for this disease include lung transplantation and two medications that slow - but do not stop - fibrosis progression^2^. One major barrier to the development of efficacious therapies for IPF has been a knowledge gap regarding the cellular and molecular mechanisms of IPF fibrosis progression^3,4^.

We previously identified mesenchymal progenitor cells (MPCs) from human IPF lung tissue that serve as cells of origin for IPF fibroblasts^5^. These cells exhibited canonical MPC properties including: (1) tri-lineage differentiation potential; (2) characteristic cell-surface markers; and (3) anchorage- independent colony formation. In contrast to lung MPCs from patient-controls, IPF MPCs displayed the following durable (i.e., cell-autonomous) pathological features: (1) greater anchorage-independent colony formation; (2) generated daughter fibroblasts with the characteristic IPF signaling signature that produce fibrotic lesions in zebrafish and mouse xenografts; and (3) caused interstitial lung fibrosis in humanized mice. Bulk RNA sequencing of MPCs from IPF patients compared to patient-controls revealed significant transcriptomic differences including the expression of genes governing cell proliferation and gene expression itself. Histological analysis of primary human IPF lung samples identified MPCs at the mitotically active perimeter region of the fibroblastic focus, adjacent to relatively preserved alveolar walls^5^. Thus, the IPF MPC population harbors a cell-autonomous pathological phenotype.

Data from several studies indicate that mesenchymal cells, including MPCs, represent a heterogeneous population. Recent studies have identified lung mesenchymal cell subgroups with unique roles in airway maintenance and repair related to their specific location and cross-talk with epithelial cells^6,7^. MPC properties are dependent on multiple factors including site of origin^8,9^, gender^10^, and environmental cues^5^. Among the IPF lung MPC population we observed significant cell-to-cell variability in their colony forming capacity^5^. Based on this evidence, we hypothesize that heterogeneity exists amongst the MPC population.

Single-cell RNA sequencing has emerged as a powerful tool for detecting heterogeneity in a population of cells. A fundamental challenge with single-cell sequencing experiments is organizing the cells in an unbiased and biologically relevant fashion such that differences between groups or across a spectrum can be elucidated with the minimum number of *pre-hoc* assumptions^11^. Transcriptomic network entropy is a metric with these properties. Its underlying assumption is that an undifferentiated cell (e.g., stem cell, progenitor cell) exists in a state of transcriptomic promiscuity in its expression of cell signaling networks, such that it is poised to respond to relevant environmental cues instructing its differentiation trajectory^12^. As cells differentiate, they up-regulate pathways relevant to their ultimate biological function and down-regulate irrelevant pathways^12^. Network entropy captures this concept by quantifying the variability in the expression of pathways as defined by literature curated protein-protein interaction networks. The network entropy algorithm (Single-Cell Entropy, SCENT) has been validated to accurately reflect differentiation trajectories using single-cell RNA sequencing data in an unbiased manner that is robust to sequencing coverage and drop-out rate^13^. Given these properties of the SCENT algorithm (few *pre-hoc* assumptions, relatively unbiased, biologically validated, and biologically relevant) it represents a powerful tool for understanding heterogeneity within single-cell sequencing experiments.

To investigate the transcriptomic underpinnings of lung MPC heterogeneity we performed single-cell sequencing of lung MPCs, employing preparative procedures identical to those used in our previously published studies^4,14^. Control and IPF lung MPCs exhibited a spectrum of differentiated states with the least differentiated IPF lung MPCs displaying the largest differences from control MPCs. We identified CD44 and MARCKS as gene products uniquely identifying the most undifferentiated IPF MPCs, and localized these cells in the IPF lung to the highly cellular perimeter region of the fibroblastic focus.

## Materials and Methods

### Study subjects

Human lung tissue was procured and de-identified by the University of Minnesota Clinical and Translational Science Institute (CTSI) Biological Materials Procurement Network (BioNET).

#### Isolation and culture of primary human lung fibroblasts

To ensure comparability of the data with previously published findings regarding the lung MPC population, we utilized identical isolation and culture techniques. All studies used primary human lung fibroblasts isolated as previously described^15^ from human lung tissue including IPF explant specimens (n = 3, all tissue confirmed to fulfill diagnostic criteria for IPF including pathological diagnosis of usual interstitial pneumonia) or control (n = 3, all cancer adjacent tissue). The use of cancer adjacent tissue for patient-control fibroblasts enabled us to age match to the demographics of IPF and provided cells with a background of chronic, non-fibrotic lung disease. All tissue was verified to be tumor free by a pathologist. After isolation of fibroblasts, cells were cryopreserved until use. All cell lines were analyzed between passages two and six to minimize confounding signal due to replicative changes.

#### FACS sorting and isolation of lung MPCs

Primary cells were thawed and cultured for 14 days, stained with anti-SSEA4 Alexa Fluor 647 (AF647), and flow sorted. SSEA4^hi^ cells isolated with this approach have been shown to demonstrate a mesenchymal progenitor cell phenotype (hereafter referred to as mesenchymal progenitor cells, “MPCs”)^16^. MPCs were submitted to the University of Minnesota Genomics Center for single cell isolation and library preparation.

#### Single cell sequencing

MPCs were stained for viability and loaded into the Fluidigm C1 large cell integrated fluidic circuit (IFC). Cell lysing, reverse transcription and cDNA amplification was performed on the C1 auto-prep IFC per the manufacturer’s protocol. Libraries were constructed using the Nextera XT DNA Sample Preparation Kit, according to the manufacturer’s recommendations. Sequencing was performed on Illumina MiSeq Sequencer by 75 bp paired-end sequencing.

#### Data quality control and read alignment

Sequence quality of each library was assessed using the FastQC program, and libraries with low data quality were excluded from downstream analysis. Reads were trimmed using the trimmomatic program^17^ to remove low quality bases. Reads were aligned to the human genome (GRCH38.84) using the HISAT2 algorithm^18^. Following alignment, the number of aligned reads vs number of unmapped reads per cell was plotted and outliers (those with very high unmapped read percentage) were removed from downstream analysis. Gene abundance (mapping to Ensembl Gene IDs) was estimated using HTSeq^19^.

### Data analysis

Data were analyzed in R using publicly available packages. For all downstream analyses, data were log base 2 converted. Network Entropy was calculated using the SCENT algorithm to order cells based on differentiated state in an unbiased and biologically relevant fashion. For the SCENT algorithm, abundance estimates were linked to the Entrez Gene ID using biomart and the protein-protein interaction network was obtained from Github as part of the SCENT algorithm. SCENT analysis included the 10000 most variable genes. Gene Ontology analysis was performed using GOrilla^20^ and ToppGene^21^. Linear modeling and mixed-linear modeling was performed with R packages *lm* and *lme4*, respectively. Comparative data sets were obtained from the gene expression omnibus (GSE75748 and GSE72056). Human embryonic stem cell and neural progenitor cell data was extracted from GSE75748, and terminally differentiated cells including T-cells, B-cells, NK-cells, macrophages and endothelial cells (but not cancer-associated fibroblasts) were extracted from the melanoma dataset in GSE72056.

### Immunohistochemistry/Immunofluorescence

Fixed human lung IPF tissue samples (individual or combined in TMAs) underwent serial sectioning and were stained with Hematoxylin-Eosin, or probed with the following antibodies: anti-SSEA4 (Biolegend, #330401, 1:50), anti-CD44 (Abcam, ab101531, 1:500), anti-MARCKS (Novus, NB110-58875SS, 1:500), anti-pro-collagen I (Abcam, ab64409, 1:500). Slides were counterstained with hematoxylin. For immunofluorescence, secondary antibodies included anti-Mouse Alexa Fluor 488 (Invitrogen, A11029, 1:1000) and anti-Rabbit Alexa Fluor 594 (Invitrogen, A11072, 1:1000).

### Colony forming assays

We used FACS as outlined above to generate single cell suspensions of IPF MPCs stratified by CD44 expression (top 4% and bottom 4%). Cells were incorporated into methylcellulose gels (STEMCELL Technologies) and maintained in MSC SFM CTS (Thermo Scientific/Gibco; 37 °C, 5% CO_2_) for 7 days. Colony number was quantified microscopically and colony size was quantified with Image J.

### Study approval

All experiments utilizing patient-derived cell-lines were approved by the University of Minnesota Institutional Review Board for Human Subjects Research (IRB# 1504M68341). Written informed consent was obtained from participants prior to inclusion in the study. All methods were carried out in accordance with relevant guidelines and regulations.

Please refer to the supplement for additional detailed Materials and Methods.

## Results

**Figure 1:**
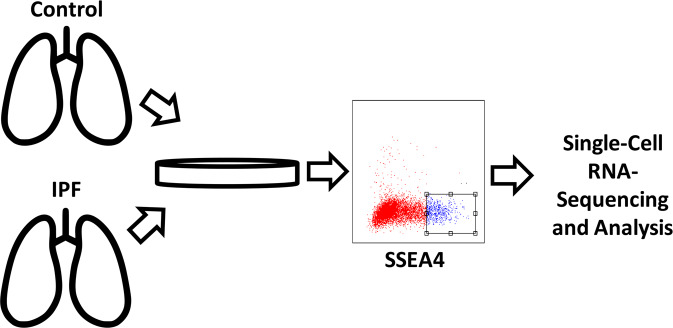
Overview of experimental Design. Primary mesenchymal cell populations were isolated from control (N = 3) and IPF (N = 3) human lung tissue and used between passages 2 and 6. FACS was used to isolate a single cell suspension of SSEA4^hi^ cells (hereafter referred to as MPCs). Cells were subjected to live/dead assessment, microscopic evaluation to ensure single cells in each chamber and sequenced on the Fluidigm C1 platform according to the manufacturer’s protocol.

We have previously shown that cells isolated from primary lung tissue explants following FACS for the SSEA4^hi^ population displayed an MPC phenotype including appropriate cell surface marker expression and tri-lineage differentiation potential^5^. In contrast to MPCs from control lungs, IPF MPCs had a durable, cell-autonomous pathological phenotype that included a distinct transcriptome and the ability to produce interstitial lung fibrosis in humanized mice. We hypothesized that MPCs isolated from the IPF lung would exhibit heterogeneity, and that we could elucidate this heterogeneity on the basis of their transcriptome. We analyzed lung MPCs from six patients (three control patients and three patients with IPF) isolated in an identical manner to that previously described^14^. After a quality-control assessment, we conducted single cell RNA sequencing using the Fluidigm C1 platform. Cells were filtered based on the number of reads, percentage of reads mapped, and quality of reads. This resulted in 335 cells (159 IPF and 176 control), which were included in downstream analyses. The experimental setup is summarized in Fig. 1, and the sequencing characteristics for each sample are summarized in supplementary table 1. The data for this publication is deposited in the BioProject repository under accession number PRJNA641647.

To investigate for consistency with our previous results, we sought to determine whether the averaged gene expression values determined by single-cell sequencing correlated with that determined by previously generated bulk RNA-sequencing of lung MPCs from control and IPF patients^5^. We compared the mean expression of all genes detected in both the single cell sequencing and bulk RNA sequencing experiments (n = 12,102 genes, supplementary Fig. 1). We determined the correlation by Spearman rank correlation to the bulk sequencing expression for both the IPF (rho = 0.72, p < 2.2e–16) and the control (rho = 0.74, p < 2.2e–16) conditions. This correlation was consistent with that previously published with correlation coefficients for individual genes ranging anywhere from 0.7 to 0.85^22^. Thus, we found acceptable agreement between bulk and single cell data.

### SSEA4^hi^ cells exhibit an MPC to fibroblast differentiation spectrum

**Figure 2:**
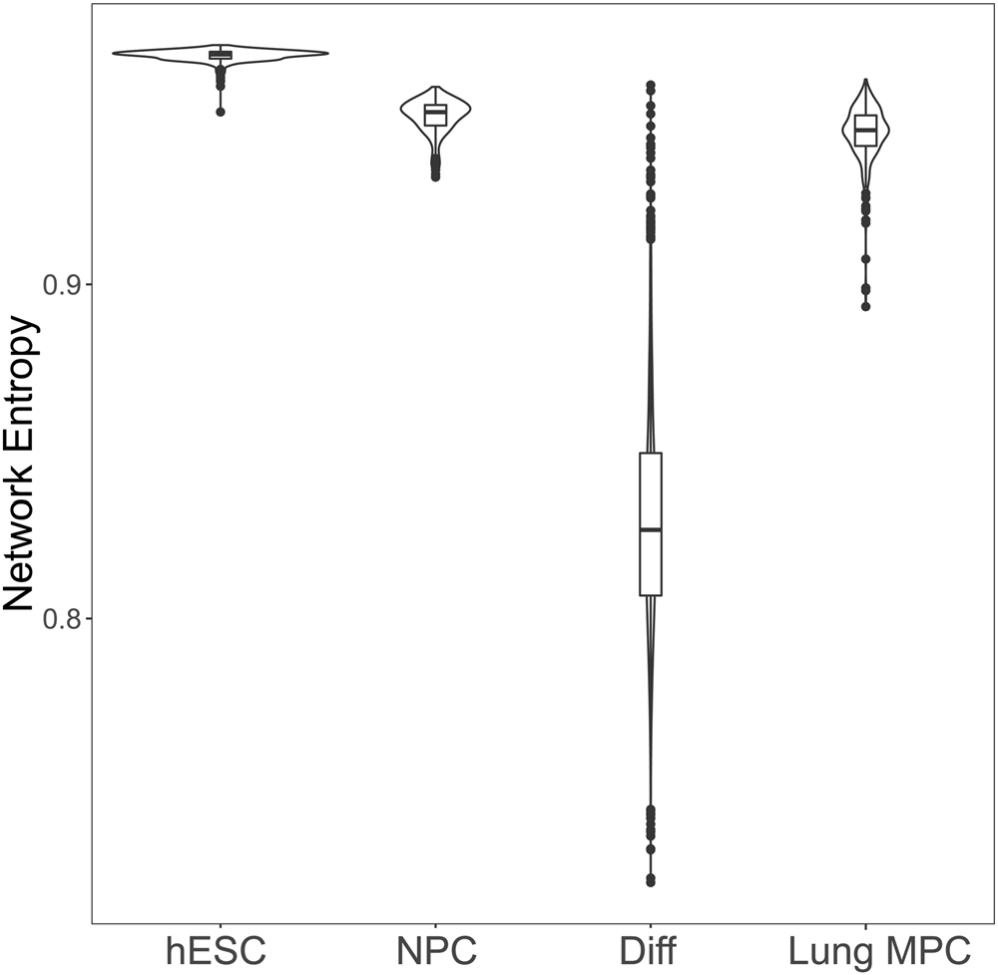
Network entropy of lung MPCs compared to other cell populations of known differentiation status. Single cell sequencing data for human embryonic stem cells (hESC), neural progenitor cells (NPC), and differentiated cells (Diff) were from the gene expression omnibus (https://www.ncbi.nlm.nih.gov/geo/). Network entropy was calculated for cells from these datasets and compared to IPF and control MPCs considered as a single group (Lung MPC).

To characterize MPCs in an unbiased manner, we utilized the Single Cell Entropy (SCENT) algorithm, which is one method to quantify the differentiated state of each cell^23^. This algorithm estimates network signaling entropy as a proxy for differentiated state, using literature curated protein-protein interaction databases to calculate a normalized signaling entropy value between zero and one. Using this approach, stem cells manifest the highest network entropy (i.e., have the least amount of cell type specific gene expression) and differentiated cells manifest the lowest network entropy (i.e., express mainly genes typical of its differentiated state)^12^. In order to calibrate our results, we calculated the network entropy of human embryonic stem cells (n = 374)^24^, neural progenitor cells (n = 173)^24^ and differentiated cells (n = 2,826)^25^ (data obtained from gene expression omnibus GSE75748 and GSE72056). Note that this cross-study analysis was conducted to determine whether we could reproduce the analysis performed in the initial description of the SCENT package^23^. Violin plots of this calculation (Fig. 2) showed that lung MPCs have a network entropy similar to that of another progenitor cell type (i.e., neural progenitor cells), and between embryonic stem cells and terminally differentiated cells (p < 2.2e–16, Mann-Whitney U-test) supporting our prior data that these cells are indeed progenitor cells. Lung MPCs also displayed a narrow network entropy distribution similar to the other stem/progenitor cell types investigated, in contrast to the broad distribution of terminally differentiated cell types. This result showed that human lung derived MPCs share transcriptomic entropy characteristics with other progenitor cell types.

We next sought to determine whether single-cell network entropy values correlated with transcriptional characteristics expected of lung derived MPCs. We performed linear regression analysis, controlling for donor identity using a linear model whereby gene expression was dependent upon network entropy and donor identity. This analysis revealed 4,586 genes either positively or negatively correlated with network entropy (at a false discovery rate of 0.05 via q-value analysis^26^). Gene ontology analysis of all correlated genes using ToppGene^21^ revealed a significant enrichment of GO functions involved in cell cycle and mitosis. We investigated the expression pattern of canonical myofibroblast genes and as expected found that many, including COL1A2, Fibronectin, and MMP11, showed increasing average expression as network entropy decreased (negative correlation with entropy).

**Figure 3:**
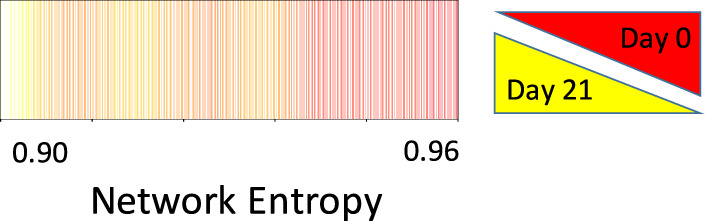
Decreasing network entropy correlates with fibroblast differentiation. Single cell expression patterns were assessed for similarity to bulk sequencing of primary human MPCs (and their progeny) from control and IPF patients (averaged together). For the bulk data, cells were analyzed immediately post-FACS (Day 0, i.e. MPCs), or after being allowed to proliferate and differentiate under standard tissue culture conditions for 21 days post-FACS isolation (Day 21, i.e. differentiated fibroblasts). Shown is the log base 2 ratio of the Spearman rank correlation of each individual cell to day 0 and day 21 cells. Cells are ordered based on network entropy. Data was smoothed using a Gaussian smoothing algorithm in R.

Given the trend for increased canonical myofibroblast gene expression across decreasing network entropy, we sought to determine whether single cell transcriptomes showed a trend of increasing similarity to myofibroblast transcriptomes with decreasing network entropy. To investigate this, bulk RNA sequencing data from freshly isolated MPCs and their differentiated progeny (i.e. fibroblasts) were downloaded from the gene expression omnibus (GSE97038)^5^. Replicate samples were averaged to define a reference transcriptome from the bulk sequencing data. Single cell transcriptomes were filtered to only include those genes that were identified in bulk sequencing datasets and were compared to both MPC and progeny transcriptomes via Spearman rank correlation. The log of the ratio of the correlation coefficients for each single cell to its reference transcriptome was then calculated. Shown in Fig. 3 is a heatmap of the log-ratios, with cells ordered according to network entropy. A Gaussian smoothing algorithm was used to display the underlying trend. Cells exhibited a spectrum of increasing similarity to the progeny gene expression pattern with decreasing network entropy. Taken together, these results support a model in which the highly entropic (i.e., least differentiated) cells show altered proliferation; whereas the less entropic (i.e., more differentiated) cells displayed transcriptomic features consistent with differentiation down a fibroblast lineage.

### Disease status is a major determinant of MPC transcriptomes

**Figure 4:**
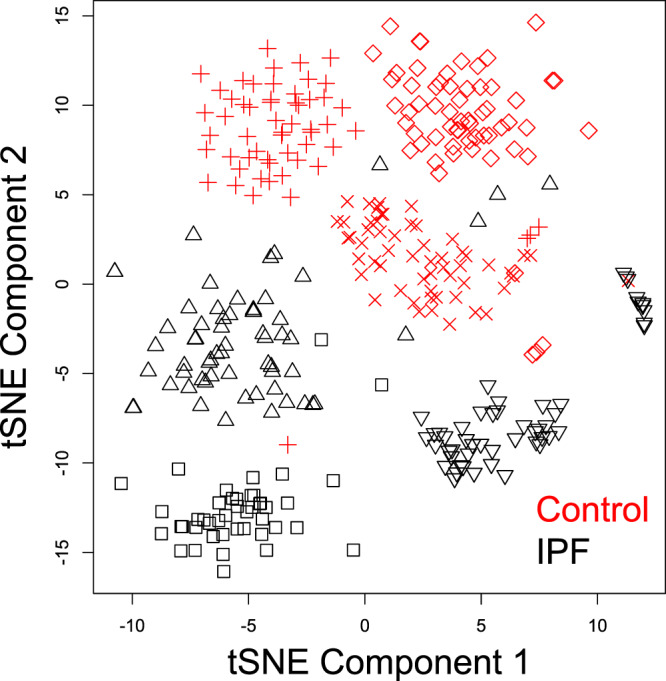
t-SNE analysis of MPCs. We performed dimensional reduction analysis using the t-distributed Stochastic nearest neighbor embedding (t-SNE) algorithm including the 10,000 most variable detected genes. Individual donors are represented by different symbols, and colors indicate donor disease status (red = Control, black = IPF).

**Figure 5:**
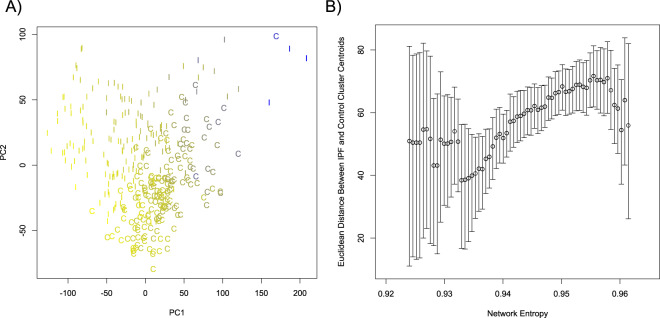
Principle component analysis (PCA) of MPCs. (**A**) We performed PCA using the prcomp algorithm in R, based on the 10,000 most variable, detected genes. Plotted are the first two principle components from the analysis. MPCs from IPF and control donors are identified (I = IPF, C = Control). Each point is colored to denote its relative network entropy (yellow = high entropy, blue=low entropy). (**B**) Euclidean distances between the centroids of the IPF and Control cell clusters were calculated across the network entropy spectrum. Cells were analyzed in a sliding boxcar fashion, with the width of the boxcar including approximately 25% of the observed network entropy range, and with steps of 1/100^th^ of the observed network entropy range. Error bars represent the 95% confidence interval, calculated using a bootstrap approach. A weighted first order fit has a positive slope that is non-zero (p-value < 2.2 × 10^−16^).

We next performed dimensional reduction analysis to examine the relationships among the IPF and control lung MPCs. We performed t-Distributed Stochastic Neighbor Embedding (t-SNE)^27,28^ as well as principle component analysis on log-transformed TPM expression values, including the top 10,000 most variable genes among all those expressed in at least one cell (n = 27,061 ensembl gene IDs meeting this criteria). Inspection of the t-SNE plot (Fig. 4) and PCA plot (Fig. 5a) revealed two levels of organization. First, on the t-SNE plot and PCA plots, cells were grouped primarily by disease tag (IPF vs Control), indicating that the donor’s disease status is a primary parameter distinguishing these cells. Second, on the t-SNE analysis we observed that cells clustered according to donor identity. Overlaid on the PCA plot in Fig. 5a is the network entropy of each cell with blue designating the lowest network entropy and yellow designating the highest network entropy. We also performed PCA analysis on a drop-out rate corrected dataset using the CIDR algorithm and this revealed similar results (supplementary Fig. 2). Visual inspection of these plots suggested that the greatest separation between IPF and control MPCs occurred amongst the most highly entropic cells. In order to rigorously test this observation, we calculated the Euclidean distance in the principle component 1 and 2 space between the centroid of the IPF and Control cell clusters across the entropy spectrum using a sliding boxcar approach, and estimated the error in this distance metric by bootstrapping. There was a significantly positive trend to the inter-centroid distance as a function of network entropy (Fig. 5b). This shows that MPCs from control and IPF tissue have increasingly discernible transcriptional profiles with increasing network entropy. Taken together, these data show that disease status as well as donor identity are major determinants of the MPC transcriptome, and that the divergence between control and IPF cells increases with increasing network entropy (i.e. the least differentiated cells differ the most).

In order to investigate for systematic biases in the network entropy calculation, we tested for an influence of read depth as well as cell cycle phase on network entropy. Although network entropy has been previously shown to be relatively insensitive to sequencing depth, given the reliance of our analysis on network entropy, we sought to ensure that read depth did not significantly confound our network entropy calculations. We compared read depth to the first 10 individual principle component loadings from the PCA analysis, and found that no individual principle component was correlated with read depth until principle component 6 (Spearman correlation coefficient of −0.32, bonferonni corrected p-value 3.4 × 10^−9^). We found that principle component 6 explained only 0.39% of the variability of our data, and thus did not pursue correction for read depth in downstream analyses. Network entropy has previously been shown to not be biased by cell cycle phase^13^. In order to ensure that this held true for our analysis, we calculated G1S and G2M cell cycle phase scores as previously described^13,29^. These scores reflect the average number of standard deviations away from the mean for genes involved in a particular cell cycle, such that the higher the score the more likely a cell is in that phase of the cell cycle. No cell had a G1S or G2M score outside of the range −1 to 1 (supplementary Fig. 3). Reassuringly, there was no bias for a higher cell cycle phase score based on network entropy. This is consistent with previous findings that the network entropy value is not biased towards a particular cell cycle phase^13^. In total, we did not identify systematic bias in the network entropy calculation either related to read depth or cell cycle phase.

To define gene interactions involved in the differentiation of lung MPCs, we utilized network analysis for identification of genes driving expression changes across the differentiation spectrum. We utilized the SCODE algorithm^30^ which infers networks from single cell sequencing data across a differentiation trajectory. In this analysis we utilized network entropy as a surrogate for the degree of differentiation (i.e. the location along the differentiation trajectory), corrected the data for patient ID in order to isolate gene expression changes across different cells using linear modeling in R, and included the 1000 genes with the highest variability. Shown in Table 1 are the 10 most highly connected genes from this analysis. Here, connectivity for a given gene refers to the number of other genes whose expression pattern is correlated with it across the differentiation spectrum, with high connectivity genes likely to be drivers of differentiation-dependent transcriptome changes. Several of these genes (e.g. AURKB) have previously been shown to play a role in pluripotent cell biology and in promoting phenotypes associated with IPF mesenchymal cells^31–37^.

**Table 1:** Most Highly Connected Hub Genes in SCODE Analysis.

	Gene Symbol	Gene Name	Connectivity
1	FHL2	Four and a Half Lim Domain Protein	413
2	PCID2	PCI Domain Containing 2	399
3	USP4	Ubiquitin Specific Peptidase 4	396
4	CDH13	Cadherin 13	338
5	SUSD6	Sushi Domain Containing 6	336
6	AURKB	Aurora Kinase B	332
7	SEMA3A	Semaphorin 3 A	331
8	CSE1L	Chromosome Segregation 1 Like	331
9	KIF20A	Kinesin Family Member 20 A	321
10	METTL22	Methyltransferase Like 22	319

### Comparison of network entropy between IPF and control MPCs

We next sought to determine whether MPCs derived from IPF and control patients displayed different network entropy (Supplementary Fig. 4). To account for the patient ID-derived signal noted in the dimensional reduction analyses shown above (Figs. 4 and 5), we utilized a mixed-model approach where disease status (IPF vs Control) was treated as a fixed variable and a random patient-specific effect term was included. Using this approach, we did not find a significant difference in network entropy in IPF versus Control MPCs (χ^2^ = 2.44, p = 0.12).

### Network analysis reveals an IPF-MPC specific differentiation signature

In order to discern the genes driving the transcriptome changes across the differentiation spectrum in IPF MPCs, we again utilized the SCODE algorithm on the patient ID corrected dataset to identify an IPF-specific gene interaction network. We first performed a SCODE analysis separately on the IPF and control MPC populations, as described above. This resulted in separate association matrices for IPF and Control MPC populations. We next subtracted the two association matrices to determine the IPF-specific association matrix^38^. In principle, elements in the association matrix that either strengthened in absolute terms or changed sign in IPF compared to control MPCs were subtracted to determine the change in association. Interactions that weakened in absolute terms in IPF compared to control conditions were set to zero^38^. Using this approach, we rank-ordered genes based on their connectivity in the IPF-specific network, with the top 10 connected genes shown in Table 2. We performed gene ontology enrichment analysis on this rank-ordered IPF specific gene list using GOrilla^20^. Of the 19 ontologies found to be significant with a false discovery rate <0.05 based on q-value analysis, we found 18 ontologies related to the regulation of mitotic cell cycle and one ontology related to regulation of the MAPK cascade (supplementary table 2).

**Table 2:** Most Highly Connected IPF-Sepcific Hub Genes in SCODE Analysis.

	Gene Symbol	Gene Name	Connectivity
1	RAB22A	RAS-Related Protein RAB-22A	380
2	SUSD6	Sushi Domain Containing 6	374
3	CDC20	Cell-Division Cycle Protein 20	370
4	C1orf198	Chromosome 1 Open Reading Frame 198	349
5	SCARA3	Scavenger Receptor Class A Member 3	341
6	IGFBP3	Insulin Like Growth Factor Binding Protein 3	327
7	CCL2	C-C Motif Cheomkine Ligand 2	318
8	USP4	Ubiquitin Specific Peptidase 4	311
9	METTL22	Methyltransferase Like 22	307
10	NRIP3	Nuclear Receptor-Interacting Protein 3	288

We next looked for upstream hub molecules influencing gene expression patterns. We utilized ingenuity pathway analysis to query previously annotated interaction networks to identify regulatory factors that were enriched in annotated associations with our IPF specific gene list. The top 5 identified upstream regulators included ERBB2, TP53, TGFB1, CDKN1A, and EGFR; a result consistent with a strong fibrosis signal for IPF MPCs.

### Identification of pathological MPCs in IPF lung tissue

**Figure 6:**
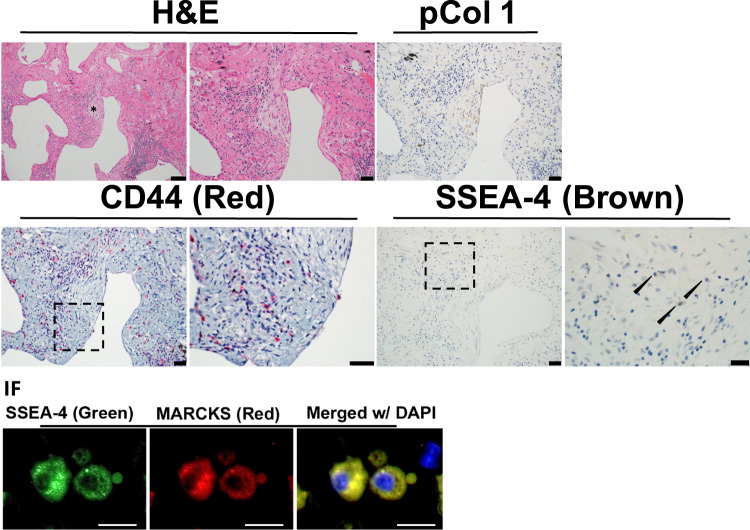
Cells expressing CD44, SSEA-4 and MARCKS reside in the cellular perimeter region of the fibroblastic focus. An Idiopathic Pulmonary Fibrosis (IPF) specimen was serially sectioned at 4 μm and processed for histology, immunohistochemistry (IHC) and immunofluorescence (IF). IHC: Representative images for Hematoxylin and Eosin (H&E) staining (scale bar represents 50 μm left and 20 μm right) with an asterisk labeling a fibroblastic focus; Immunostaining for anti-procollagen type I (brown, scale bar 20 μm); anti-CD44 (red, scale bar 20 μm, dashed outline box, scale bar 20 μm); anti-SSEA4 (brown, scale bar 20 μm, dashed outline box, scale bar 10 μm). Lower panel: Immunostaining anti-SSEA-4 (green), MARCKS (red), DAPI (blue, scale bar 20 μm). A small apoptotic body is noted adjacent to the cell on the right. Immunofluorescence images obtained at the perimeter of the fibroblastic focus.

To assess the clinical relevance of our single cell sequencing data, we conducted a morphological analysis to localize the highly entropic MPCs in IPF lung tissue. We first sought to identify distinguishing transcriptomic features of the most highly entropic IPF MPCs by performing linear modeling in R such that gene expression was modeled as a function of entropy, donor identity, disease tag, as well as a cross term between entropy and disease tag. We selected all genes with a significant cross term such that there was a greater slope with increasing entropy in IPF vs Control cells (using FDR <0.05 as a significance threshold). We found 148 genes matching this criterion, making them potential biological markers for the highly entropic IPF MPCs (supplemental table 3). Inspection of this list revealed two gene products, CD44 and MARCKS, whose expression profile was predicted to identify highly entropic IPF MPCs. We performed histological analysis of IPF tissue to identify cells expressing SSEA4 (i.e. MPCs), CD44, and MARCKS. TMAs were serially sectioned, and stained with H&E, CD44, SSEA4, SSEA4/MARCKS dual-immunofluoresence, and human procollagen I. Fibroblastic foci were identified as regions with a procollagen I positive core region and a perimeter cellular region. A representative fibroblastic focus is shown in Fig. 6, with additional foci from the same patient shown in supplementary Fig. 5, and foci from two additional patients shown in supplementary Fig. 6A,B. High resolution images from Fig. 6 are available for review along with our supplemental information. Cells in the core of the focus express procollagen I^39^, bounded by highly cellular perimeter regions^3^. Consistent with our previous results, we identified cells expressing SSEA4 within the perimeter region of the focus^5^. Additionally, we found that the CD44 positive cells co-localized with SSEA4 within the perimeter region of the focus. A subset of cells co-expressed SSEA4 and MARCKS. While a quantitative analysis of the co-localization of these markers cannot be conducted with the serial sectioning approach used here, we qualitatively found that SSEA4, MARCKS and CD44 co-localized to the perimeter region of the IPF fiboblastic focus. These findings indicate that the most highly entropic MPCs reside within the cellular perimeter region of the IPF fibroblastic focus, placing them in the correct anatomic location to fulfill a pathogenic role in disease progression.

### CD44^hi^ IPF MPCs exhibit greater colony forming capacity than CD44^lo^ IPF MPCs

**Figure 7:**
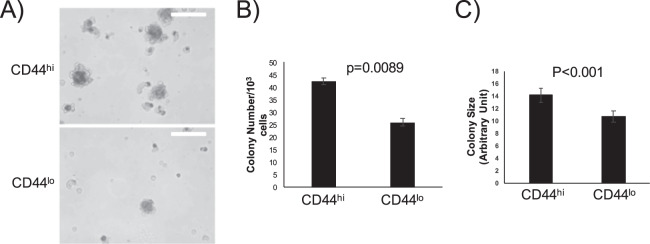
CD44^hi^ IPF MPCs exhibit greater colony forming capacity. IPF MPCs were incorporated into methylcellulose gels and cultured for 7 days. (**A**) Examples of colony images (white bar = 100 micrometers). (**B**) Colony number. (**C**) Colony size. P-values calculated with unpaired student t-test.

Our bioinformatics and histological analyses predict that the most highly entropic IPF MPCs, marked by high expression of the cell surface marker CD44, will exhibit progenitor cell properties. A defining feature of stem/progenitor cells is the capacity to form colonies in an anchorage-independent manner. To test this prediction, we used FACS to separate IPF MPCs into CD44^hi^ and CD44^lo^ fractions, which represented the top and bottom 4% of CD44 expression, respectively. These freshly sorted cells were seeded onto methylcellulose gels and colony number and size were quantified after one week in culture. As shown in Fig. 7, the CD44^hi^ IPF MPC population formed more colonies with a larger average colony size. These results confirm our bioinformatics prediction that the IPF MPCs with highest network entropy would exhibit the greatest colony forming capacity.

## Discussion

Through single-cell RNA sequencing and bioinformatics analysis of MPCs derived from control and IPF lung tissue, we found that IPF MPCs represent a heterogeneous population with the least differentiated MPCs displaying the greatest distinction from control MPCs. Using this transcriptomic signature as a guide, we identified these cells at the perimeter of the fibroblastic focus, sharply demarcated from myofibroblasts residing in the fibroblastic focus core. Our data support a model in which IPF disease progression is mediated by MPCs that acquire fibrogenic properties early in their differentiation trajectory, giving rise to α-smooth-muscle actin positive myofibroblasts actively synthesizing extracellular matrix.

We have previously shown that SSEA4^hi^ lung cells have the biological characteristics of MPCs^5^. Here we showed that this population of cells has transcriptomic network entropy values between differentiated cells and human embryonic stem cells; and similar to another progenitor cell type (neural progenitor cells). Based on their transcriptome, SSEA4^hi^ cells exist along a differentiation continuum between an undifferentiated state and differentiated fibroblasts. Our data support this trajectory toward a fibroblast lineage based on upregulation of canonical ECM-related genes and comparative analysis to reference transcriptomes. Our results provide strong evidence that SSEA4^hi^ cells are MPCs capable of differentiating into lung fibroblasts.

We previously published that the IPF MPC population is enriched for pathogenic, pro-fibrotic cells capable of recapitulating many aspects of IPF in xenograft models^5^. Here we show that the MPC population exhibits a spectrum of differentiated states based on the organization of transcriptomic signatures. Dimensional reduction analysis revealed that when comparing control and IPF MPC populations, it is the most highly entropic cells that differ the most. Notably, we found that the lung MPC population exists along a spectrum of differentiated states and did not identify distinct clusters of cells. However, our data do not exclude the possibility that small subpopulations may exist that our study was underpowered to detect.

Through comparative network analysis, we showed that highly entropic IPF MPCs have a unique transcriptomic signature characterized by activation of proliferation pathways, developmental programs, and influences of P53 and TGFbeta. This finding is consistent with previous reports of both TGFbeta^40^, as well as P53 degradation in an S100A4 mediated manner^16^, having significant roles in the biology of IPF MPCs and the development of fibrosis.

Several publications have investigated mesenchymal cells from IPF or models of lung fibrosis using single cell sequencing. We investigated our data for evidence that IPF MPCs had expression characteristics of previously identified mesenchymal cell subgroups including Axin-2, Axin2-Palpha, Acta2, FGF7, Pdgfrb, LGR5, or LGR6^6,41,42^. No clear pattern of expression was detected. This likely reflects the fact that our study exclusively investigated cells harboring an embryonic determinate (i.e. SSEA4), and thus the cell population investigated here is upstream of previously identified mesenchymal cell subgroups. Future studies will be required to delineate the differentiation pathways from MPC to the previously identified mesenchymal cell populations.

We utilized histopathology of IPF lung tissue to identify the anatomic location of the most highly entropic MPCs in our cell population, marked by expression of the proteins CD44 and MARCKS. Intriguingly, CD44 has been shown to play a role in the development of pulmonary fibrosis as a regulator of fibroblast invasion^43^. Furthermore, in several cancer models, CD44 is highly expressed on cancer stem cells. Further investigation has elucidated a mechanistic role for CD44 in maintaining the cancer stem cell phenotype as well as in cancer invasion and metastasis^44^. Our lab has previously shown that hyaluronic acid, a ligand for CD44, is enriched in the fibroblastic focus. These findings suggest that the interaction between undifferentiated CD44^hi^ MPCs and hyaluronic acid in the fibroblastic focus may serve to drive progression of fibrosis in IPF. MARCKS is a target of protein kinase C that serves to catalyze actin filament crosslinking to promote cell motility and mitogenesis^45^. MARCKS has been linked to metastasis in multiple cancers^46^, and plays a role in maintaining the cancer associated fibroblast phenotype^47^. Given the body of literature on these two proteins, it is intriguing that in addition to being highly expressed in the least differentiated IPF MPCs, they may play functional roles in conferring a pathological phenotype.

A limitation of our experimental design was the focus on cell autonomous features and the loss of environmentally mediated signals. This limitation was exemplified by our inability to identify MPCs as participating in the cell cycle, despite previous *in vivo* data from IPF lung tissue showing that IPF MPCs express Ki-67. This suggests that IPF MPC cycling is likely not cell autonomous, but rather depends upon signals in the fibroblastic focus niche derived from the ECM as well as neighboring epithelial and immune cells. Additionally, our study is limited based on the comparison of cells isolated from IPF tissue to cells isolated from cancer adjacent tissue which, while histologically normal, likely has transcriptomic signatures different from normal lung. Notably, lung cancer and IPF occur in the aging lung in a chronic inflammatory context. Given this, the comparison of IPF to cancer adjacent tissue allows for isolation of the cellular response leading to progressive fibrosis, from that due solely to lung inflammation; whereas comparison of IPF to entirely normal would likely give rise to confounding signals representing both chronic underlying inflammation and progressive fibrosis.

The data presented here suggests that IPF fibroblasts acquire a pathological phenotype at the earliest stages of their differentiation. This unexpected finding begs the question of how this phenotype is acquired. Given the growing body of literature on mesenchymal cell – monocyte/macrophage crosstalk, and the co-localization of macrophages and MPCs at the fibroblastic focus perimeter, it is enticing to speculate that aberrantly activated tissue macrophages might participate in the corruption of a fraction the tissue resident MPCs. The finding that the pathological MPCs highly express the hyaluronic acid (HA) receptor CD44 raises the possibility that the acquisition of an aberrant MPC phenotype could occur indirectly through the secretion of cytokines to modify hyaluronic acid synthesis and/or degradation through cellular intermediates. These hypotheses require further experimental exploration beyond the scope of the present work

The cell-autonomous characteristics of pathological IPF MPCs we have described motivate multiple questions. One question is how IPF MPCs interact with their unique extracellular niche. We have shown that IPF MPCs reside within the mitotically active perimeter region of the fibroblastic focus, and that this region is rich in the ECM component hyaluronic acid (HA)^39^. The interaction of CD44 with HA may be an important aspect of the unique biology of IPF MPCs. A second question is how the interaction of MPCs with other cells, including epithelial and inflammatory/immune cells, within and around the fibroblastic focus drives and/or maintains a pathological phenotype. Through understanding the MPC interaction with the ECM and other cells, we hope to uncover novel therapeutic opportunities targeting this crucial cell of origin for IPF.

### Endnote

Supplementary data available at the following link: https://github.com/dbeisang/MPC_single_cell/blob/master/Supplementary%20data.pdf

## Supplementary information

Supplementary Information 1.

Supplementary Information 2.

Supplementary Information 3.

Supplementary Information 4.

## References

[1] King TE, Pardo MD (2011). A PhD, Selman M MD. Idiopathic pulmonary fibrosis. The Lancet.

[2] Adegunsoye A, Strek ME (2016). Therapeutic Approach to Adult Fibrotic Lung Diseases. Chest.

[3] Herrera J, Henke CA, Bitterman PB (2018). Extracellular matrix as a driver of progressive fibrosis. J Clin Invest.

[4] Herrera J (2018). Dicer1 Deficiency in the Idiopathic Pulmonary Fibrosis Fibroblastic Focus Promotes Fibrosis by Suppressing MicroRNA Biogenesis. Am J Respir Crit Care Med.

[5] Xia H (2014). Identification of a Cell-of-Origin for Fibroblasts Comprising the Fibrotic Reticulum in Idiopathic Pulmonary Fibrosis. The American Journal of Pathology.

[6] Xie T (2018). Single-Cell Deconvolution of Fibroblast Heterogeneity in Mouse Pulmonary Fibrosis. CellReports.

[7] Xu, Y *et al*. Single-cell RNA sequencing identifies diverse roles of epithelial cells in idiopathic pulmonary fibrosis. *JCI Insight* 2016;1:1–18. 10.1172/jci.insight.90558 PMC5135277 27942595

[8] Rolandsson Enes S (2016). MSC from fetal and adult lungs possess lung-specific properties compared to bone marrow-derived MSC. Sci Rep.

[9] Barrett A (2018). Human Wharton’s Jelly Mesenchymal Stem Cells Show Unique Gene Expression Compared to Bone Marrow Mesenchymal Stem Cells Using Single-Cell RNA-Sequencing. Stem Cells Dev.

[10] Sammour, I. *et al*. The Effect of Gender on Mesenchymal Stem Cell (MSC) Efficacy in Neonatal Hyperoxia-Induced Lung Injury. In: Kirchmair R, editor. *Plos One* 2016;11:e0164269–19. 10.1371/journal.pone.0164269 PMC5053475 27711256

[11] Kolodziejczyk AA, Kim JK, Svensson V, Marioni JC (2015). The technology and biology of single-cell RNA sequencing. Mol Cell.

[12] Banerji CRS (2013). Cellular network entropy as the energy potential in Waddington’s differentiation landscape. Sci Rep.

[13] Enver T, Teschendorff AE (2017). Single-cell entropy for accurate estimation of differentiation potency from a cell&rsquo;s transcriptome. Nature Communications.

[14] Parker MW (2014). Fibrotic extracellular matrix activates a profibrotic positive feedback loop. J Clin Invest.

[15] Larsson, O *et al*. Fibrotic Myofibroblasts Manifest Genome-Wide Derangements of Translational Control. In: Barnes PJ, editor. *PLoS ONE* 2008;3:e3220–12. 10.1371/journal.pone.0003220 PMC2528966 18795102

[16] Xia H (2017). Calcium-binding protein S100A4 confers mesenchymal progenitor cell fibrogenicity in idiopathic pulmonary fibrosis. J Clin Invest.

[17] Bolger AM, Lohse M, Usadel B (2014). Trimmomatic: a flexible trimmer for Illumina sequence data. Bioinformatics.

[18] Kim D, Langmead B, Salzberg SL (2015). HISAT: a fast spliced aligner with low memory requirements. Nat Meth.

[19] Anders S, Pyl PT, Huber W (2015). HTSeq–a Python framework to work with high-throughput sequencing data. Bioinformatics.

[20] Eden E, Navon R, Steinfeld I, Lipson D, Yakhini Z (2009). GOrilla: a tool for discovery and visualization of enriched GO terms in ranked gene lists. BMC Bioinformatics.

[21] Chen J, Bardes EE, Aronow BJ, Jegga AG (2009). ToppGene Suite for gene list enrichment analysis and candidate gene prioritization. Nucleic Acids Res.

[22] Schwalie PC (2018). A stromal cell population that inhibits adipogenesis in mammalian fat depots. Nature.

[23] Enver T, Teschendorff AE (2017). Single-cell entropy for accurate estimation of differentiation potency from a cell&rsquo;s transcriptome. Nature Communications.

[24] Chu L-F (2016). Single-cell RNA-seq reveals novel regulators of human embryonic stem cell differentiation to definitive endoderm. Genome Biology.

[25] Tirosh I (2016). Dissecting the multicellular ecosystem of metastatic melanoma by single-cell RNA-seq. Science.

[26] Storey, J. D., Bass, A. J. & Dabney, A. *qvalue: Q-value estimation for false discovery rate control*. *R package version* (2015).

[27] van der Maaten L (2014). Accelerating t-SNE using Tree-Based Algorithms. Journal of Machine Learning Research.

[28] van der Maaten L, Hinton G (2008). Visualizing Data using t-SNE. Journal of Machine Learning Research.

[29] Whitfield, M. L. *et al*. Identification of Genes Periodically Expressed in the Human Cell Cycle and Their Expression in Tumors. In: Solomon MJ, editor. *MBoC* 13:1977–2000 (2002). 10.1091/mbc.02-02-0030. PMC117619 12058064

[30] Matsumoto, H *et al*. SCODE: an efficient regulatory network inference algorithm from single-cell RNA-Seq during differentiation. In: Bar-Joseph Z, editor. *Bioinformatics* 33:2314–2321 (2017). 10.1093/bioinformatics/btx194 PMC5860123 28379368

[31] Xu Y (2018). KIAA0247 inhibits growth, migration, invasion of non-small-cell lung cancer through regulating the Notch pathway. Cancer Sci.

[32] Zhu J (2018). H19/miR‐148a/USP4 axis facilitates liver fibrosis by enhancing TGF‐β signaling in both hepatic stellate cells and hepatocytes. J Cell Physiol.

[33] Han T (2016). Identification of novel genes and networks governing hematopoietic stem cell development. EMBO Rep.

[34] Zhao X (2018). Overexpression of KIF20A confers malignant phenotype of lung adenocarcinoma by promoting cell proliferation and inhibiting apoptosis. Cancer Med.

[35] Jin J (2019). Pirfenidone attenuates lung fibrotic fibroblast responses to transforming growth factor-β1. Respir Res.

[36] Dai YEB (2014). Pcid2 Inactivates Developmental Genes in Human and Mouse Embryonic Stem Cells to Sustain Their Pluripotency by Modulation of EID1 Stability. Stem Cells.

[37] Bauer Y (2015). A Novel Genomic Signature with Translational Significance for Human Idiopathic Pulmonary Fibrosis. Am J Respir Cell Mol Biol.

[38] Taroni JN (2017). A novel multi-network approach reveals tissue-specific cellular modulators of fibrosis in systemic sclerosis. Genome Med.

[39] Herrera J (2019). Registration of the extracellular matrix components constituting the fibroblastic focus in idiopathic pulmonary fibrosis. JCI Insight.

[40] Fernandez IE, Eickelberg O (2012). The Impact of TGF-β on Lung Fibrosis. Proc Am Thorac Soc.

[41] Single-cell RNA sequencing identifies diverse roles of epithelial cells in idiopathic pulmonary fibrosis. *JCI Insight* 2016;1:621–18. 10.1172/jci.insight.90558 PMC5135277 27942595

[42] Zepp JA (2017). Distinct Mesenchymal Lineages and Niches Promote Epithelial Self-Renewal and Myofibrogenesis in the Lung. Cell.

[43] Li Y (2011). Severe lung fibrosis requires an invasive fibroblast phenotype regulated by hyaluronan and CD44. J Exp Med.

[44] Yan Y, Zuo X, Wei D (2015). Concise Review: Emerging Role of CD44 in Cancer Stem Cells: A Promising Biomarker and Therapeutic Target. STEM CELLS Translational Medicine.

[45] Sheats, M. K. *et al* MARCKS (Myristoylated Alanine-Rich C Kinase Substrate) and Lung Disease. *Am J Respir Cell Mol Biol*, rcmb.2018–0285TR–38, 10.1165/rcmb.2018-0285TR (2018).

[46] Chen C-H (2013). A peptide that inhibits function of Myristoylated Alanine-Rich C Kinase Substrate (MARCKS) reduces lung cancer metastasis. Oncogene.

[47] Yang Z (2016). MARCKS contributes to stromal cancer-associated fibroblast activation and facilitates ovarian cancer metastasis. Oncotarget.

