# Supplementary material for: Single-cell RNA sequencing reveals that lung mesenchymal progenitor cells in IPF exhibit pathological features early in their differentiation trajectory
Source: Sci Rep. 2020 Jul 7;10:11162. doi: 10.1038/s41598-020-66630-5 (PMC7341888; doi:10.1038/s41598-020-66630-5)
Supplement: Supplementary file 1 — Supplementary Information 1. [file 41598_2020_66630_MOESM1_ESM.pdf]

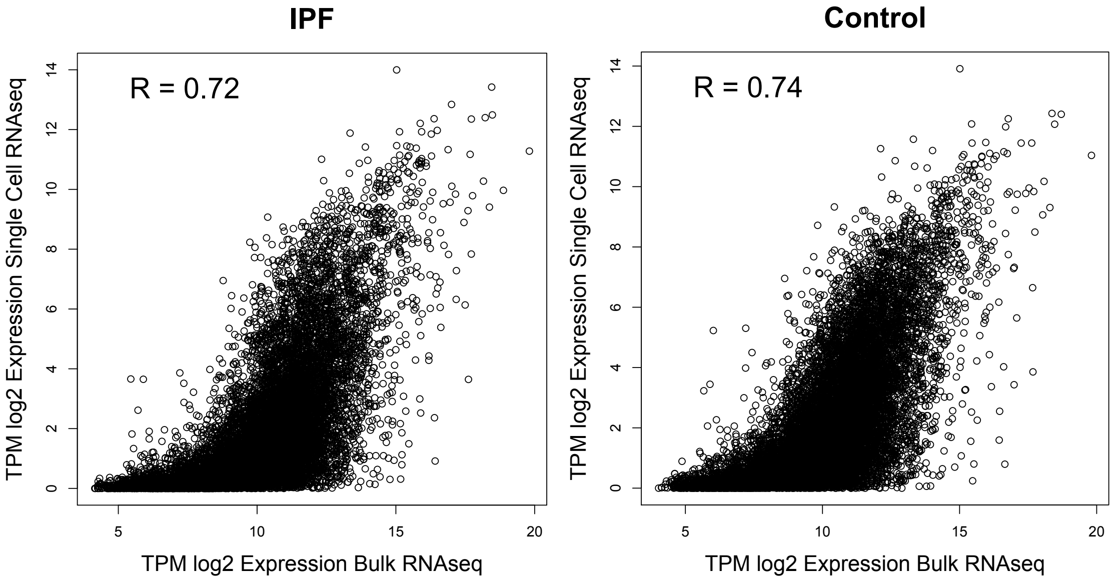

Supplementary Figure 1. Comparison of Single-cell RNA seq data with bulk RNA seq data from IPF and control SSEA4hi MPCs. The log2 adjusted TPM value of each detected gene from single cell and bulk RNA sequencing experiments from Control and IPF derived SSEA4hi MPCs is shown. An agreement between the bulk and single cell sequencing data is shown with Spearman rank correlations for IPF (r=0.72, p<2.2x10-16) and Control (r=0.74, p<2.2x10-16). A tail can be seen where lower average expression in single cell sequencing compared to bulk RNA sequencing is found for the low abundance transcripts. This likely reflects the expected lower capture efficiency of the low abundance transcripts in single cell sequencing experiments compared to bulk RNA sequencing experiments.

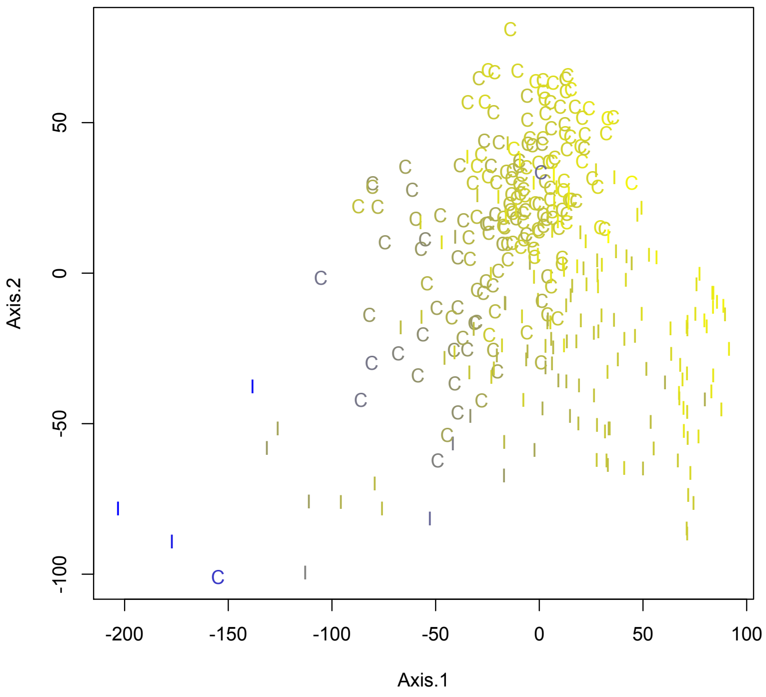

Supplementary Figure 2. PCA analysis of drop-out rate corrected data. We corrected for drop-out rate using the Clustering through Imputation and Dimensional Reduction (CIDR) algorithm. The top 10,000 most variable detected genes were included in this analysis. Plotted are the first 2 components from the PCA analysis on the imputed data. Each data point represents a cell (I=IPF, C=Control). Points are colored to indicate the network entropy of each cell (blue= lowest network entropy, yellow = highest network entropy).

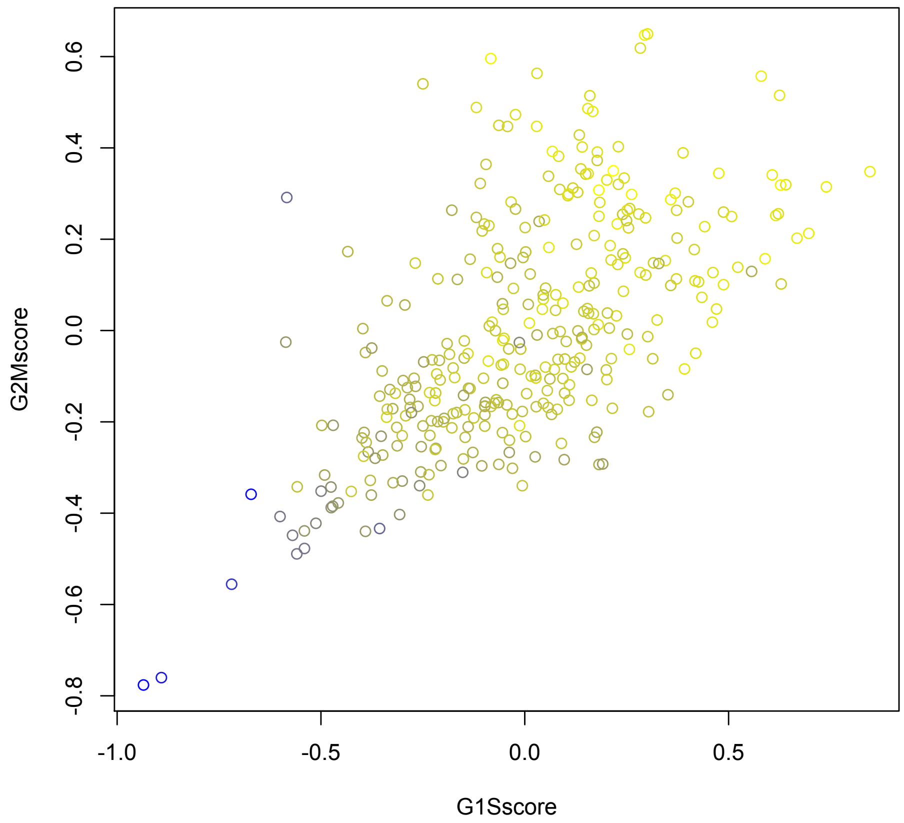

Supplementary Figure 3. Cell cycle phase score is not correlated with network entropy. Cell-cycle phase scores for G1/S and G2/M phase were calculated, and plotted for each cell. Overlayed on this plot are network entropy scores (blue= low entropy, yellow= high entropy There is no cell cycle phase bias based on network entropy.

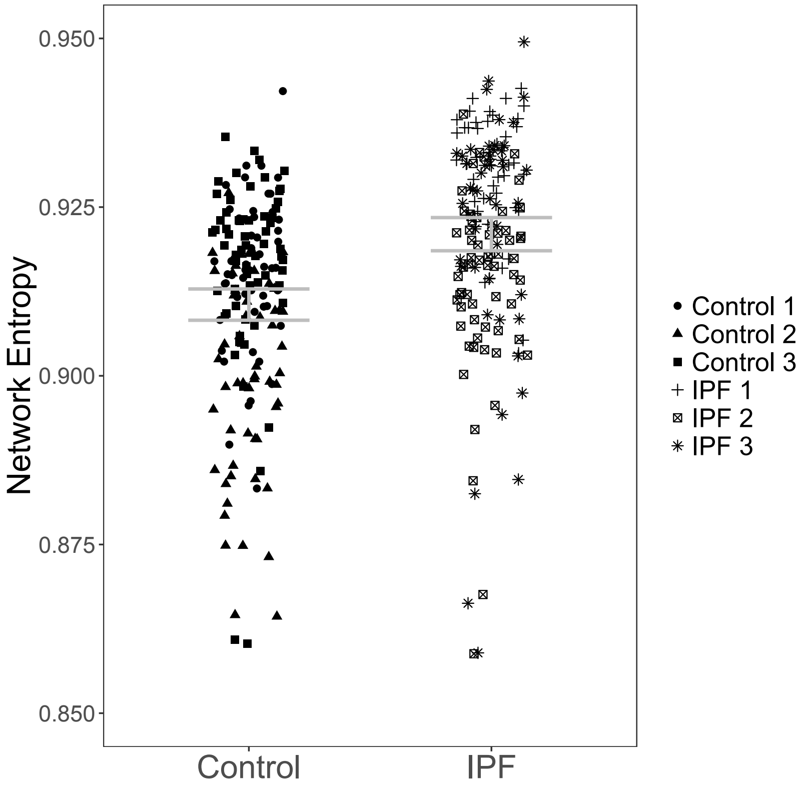

Supplementary Figure 4. IPF MPCs do not show a statistically significant difference in network entropy compared to control MPCs. We calculated network entropy for MPCs derived from control (n=3) and IPF (n=3) donors using the SCENT algorithm. Network entropy values are plotted with bars indicating the middle 2 standard deviations. IPF MPCs did not show a statistically significant difference in average network entropy compared to control MPCs (χ^2= 2.4, p=0.12).

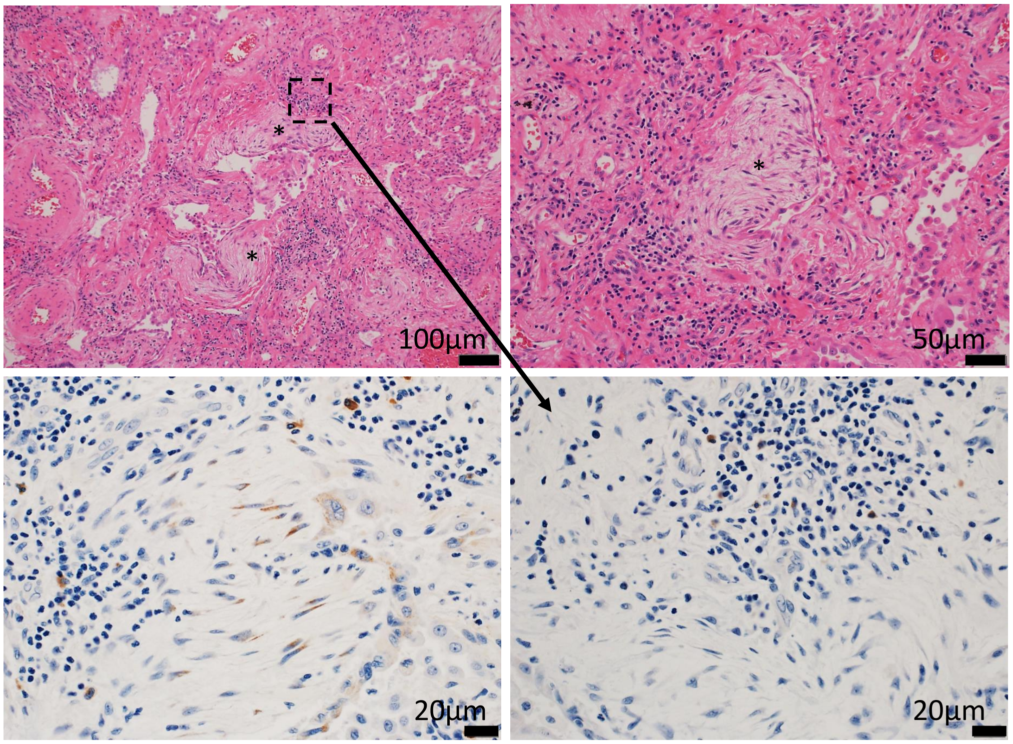

Supplementary Figure 5. IHC staining of additional fibroblastic foci identifies SSEA4 positive cells in the perimeter region. Low and high magnification views of H&E staining of two different fibroblastic foci are shown in upper left and right panels demonstrating a myofibroblast dense core region (asterisks) and adjacent highly cellular perimeter region. Procollagen-1 staining (brown) of a separate focus (lower left panel) demonstrates sharp demarcation of the fibroblastic focus core region with procollagen positive myofibroblasts. Higher magnification view of dashed region of panel upper left panel with SSEA4 staining (brown) is shown in lower right hand panel, demonstrating SSEA4 positive cells within the highly cellular perimeter region of the fibroblastic focus.

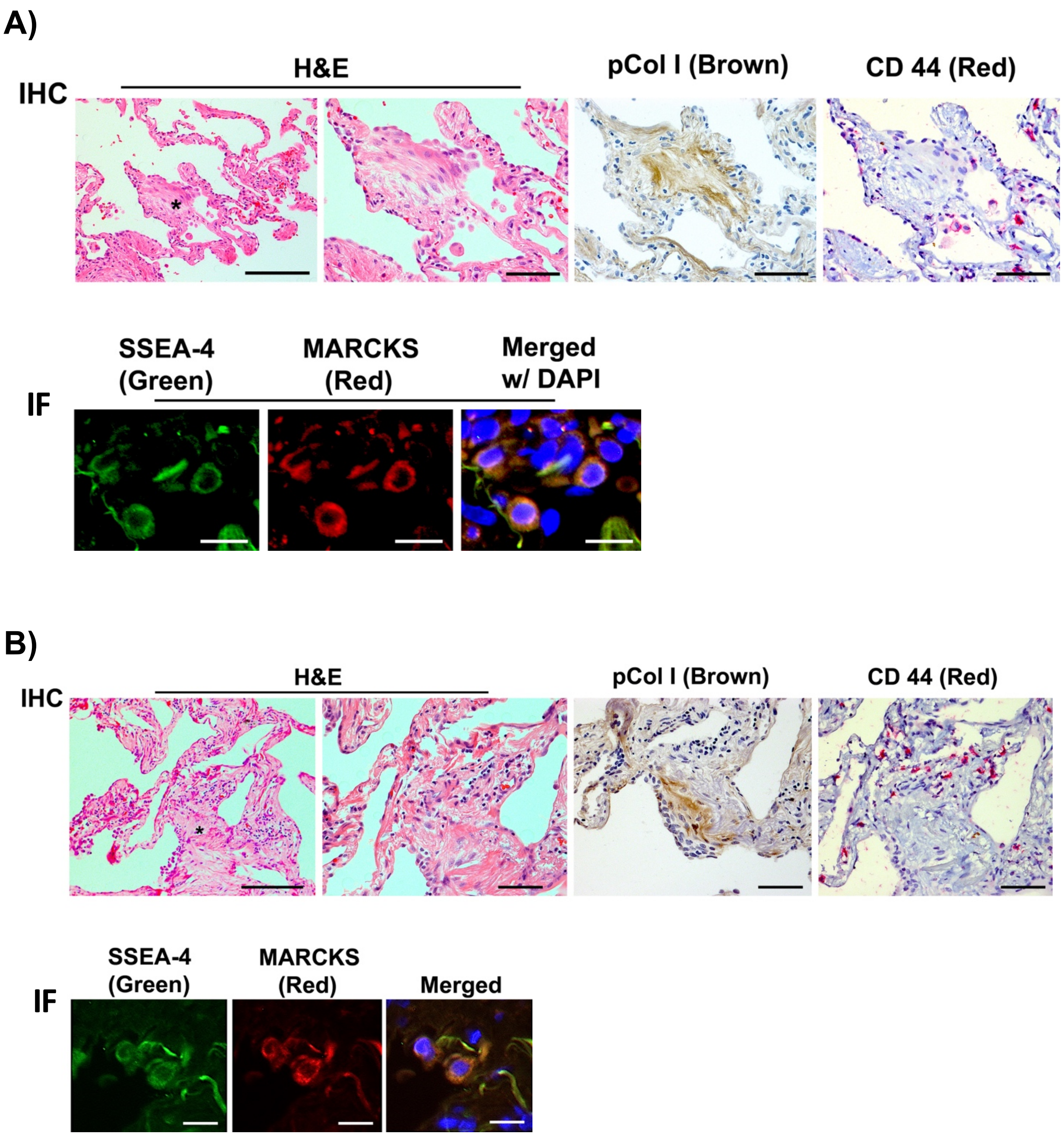

Supplementary Figure 6 Identification of highly entropic MPCs in the active front region of the fibroblastic focus in IPF lung tissue. Idiopathic Pulmonary Fibrosis (IPF) specimens from two additional patients (A and B) were serially sectioned at 4 μm and processed for histology, immunohistochemistry (IHC) and immunofluorescence (IF). Upper panel: Representative images for Hematoxylin and Eosin (H&E) staining (scale bar 400μm and 100μm) with asterisk labeling afibroblastic focus; Immunostaining for anti-procollagen type I (brown, scale bar 100μm); anti-CD44 (red, scale bar 100μm, dashed outline box, scale bar 50μm). Lower panel: Immunostaining for anti-SSEA-4 (green), MARCKS (red), DAPI (blue, scale bar 20μm).

Supplementary Table 1. Characteristics of Single Cell Sequencing.

	Control 1	Control 2	Control 3	IPF 1	IPF 2	IPF 3
**Average Number of Reads Total**	**389307**	**417136**	**440502**	**447879**	**443101**	**570528**
std dev of reads total	103539	92940	317377	186643	124654	279437
**Average Number of Reads Mapped**	**340746**	**350811**	**334393**	**393681**	**383030**	**388627**
stdev Number of Reads Mapped	92926	81460	232929	174808	108741	87579
**Average Percent Reads Mapped**	**93**	**90**	**90**	**93**	**92**	**93**
**Average # Genes Detected**	**4672**	**4796**	**3789**	**4853**	**4444**	**4796**
stdev # genes detected	630	653	695	680	696	630

Supplementary Table 2. Gene Ontology Terms Enriched in IPF-Specific Nodes

**GO term**	**Description**	**P-value**	**FDR q-value**
GO:0045787	positive regulation of cell cycle	2.94E-07	1.84E-03
GO:0051726	regulation of cell cycle	3.28E-07	1.03E-03
GO:0022402	cell cycle process	3.71E-07	7.77E-04
GO:0007346	regulation of mitotic cell cycle	4.49E-07	7.05E-04
GO:0010564	regulation of cell cycle process	5.47E-07	6.87E-04
GO:1903047	mitotic cell cycle process	1.17E-06	1.22E-03
GO:0090068	positive regulation of cell cycle process	4.21E-06	3.78E-03
GO:0045786	negative regulation of cell cycle	8.28E-06	6.50E-03
GO:0010948	negative regulation of cell cycle process	2.70E-05	1.88E-02
GO:1901990	regulation of mitotic cell cycle phase transition	2.76E-05	1.73E-02
GO:1901987	regulation of cell cycle phase transition	2.76E-05	1.58E-02
GO:0045930	negative regulation of mitotic cell cycle	4.96E-05	2.59E-02
GO:1902806	regulation of cell cycle G1/S phase transition	7.54E-05	3.64E-02
GO:2000045	regulation of G1/S transition of mitotic cell cycle	7.54E-05	3.38E-02
GO:1902807	negative regulation of cell cycle G1/S phase transition	8.47E-05	3.55E-02
GO:2000134	negative regulation of G1/S transition of mitotic cell cycle	8.47E-05	3.32E-02
GO:0043408	regulation of MAPK cascade	8.72E-05	3.22E-02
GO:0045931	positive regulation of mitotic cell cycle	1.13E-04	3.94E-02
GO:0007088	regulation of mitotic nuclear division	1.20E-04	3.95E-02
