# Supplementary material for: Single-cell RNA sequencing reveals that lung mesenchymal progenitor cells in IPF exhibit pathological features early in their differentiation trajectory
Source: Sci Rep. 2020 Jul 7;10:11162. doi: 10.1038/s41598-020-66630-5 (PMC7341888; doi:10.1038/s41598-020-66630-5)
Supplement: Supplementary file 2 — Supplementary Information 2. [file 41598_2020_66630_MOESM2_ESM.pdf]

Supplementary Materials and Methods.

Isolation and Culture of primary human lung fibroblasts. All studies used primary human lung fibroblasts isolated as previously described (1) from human lung tissue including IPF explant specimens (n=3, all tissue confirmed to fulfill diagnostic criteria for IPF including pathological diagnosis of usual interstitial pneumonia) or control (n=3, all cancer adjacent tissue). Tissue is immediately placed in isolation media (DMEM + 20% FBS + 2X antibiotic-antimycotic solution). Lung tissue is dissected into ~2-5mm cubes and placed on tissue culture plastic partially submerged in isolation media and cultured (2-3 weeks, 37^0C, 5% CO2). Explant cells were cryopreserved under liquid nitrogen until used between passages 2 and 6. All tissue was verified to be tumor free by a pathologist before a control cell line is used.

FACS and isolation of SSEA4^{hi} MPCs. Primary cells are grown for 13 to 14 days on tissue culture plastic in growth medium (DMEM + 10% FBS + antibiotic-antimycotic solution). Cells were released from dishes with trypsin/EDTA and stained with anti-SSEA4 Alexa Flour 647 (AF647). Stained cells were flow sorted with a FACSAria II P0287. Cells were separated from debris and doublets using FSC and SSC, and the resulting population was sorted to isolate the AF647 bright cells. Gating is determined using unstained and single stain controls for AF647. The SSEA4 high sub-population was submitted to the University of Minnesota Genomics Center for single cell isolation and library preparation.

Single Cell Sequencing. Sorted SSEA4hi cell suspensions were stained for viability using the LIVE/DEAD viability assay (which utilizes Calcein AM and ethidium homodimer-1 to ensure intracellular esterase activity and plasma membrane integrity) and loaded into the Fluidigm C1 large cell integrated fluidic circuit (IFC). Capture sites were microscopically examined to ensure viability and exclude doublets. Cell lysing, reverse transcription and cDNA amplification was

performed on the C1 auto-prep IFC . SMARTScribe Reverse Transcriptase was utilized for cDNA synthesis. Libraries were constructed using the Nextera XT DNA Sample Preparation Kit, according to the manufacturer's recommendations. Unique Molecular Identifiers (UMI) were not included in this workflow, and the PCR protocol utilized 21 cycles to reduce amplification error. Sequencing was performed on Illumina MiSeq Sequencer by 75bp paired-end V3 chemistry with a goal minimum of 150,000 reads per cell. The microscope used was a Nikon Ti-E.

Data Analysis: Data were analyzed in R using publicly available packages. For all downstream analysis, data were log base 2 converted. tSNE analysis was performed using the Rtsne package(2, 3), including the 10000 genes with the highest variability across all cells. Dropout rate imputation analysis was carried out using the Clustering through Imputation and Dimensional Reduction (CIDR) package using default settings (4). Network entropy was performed using the SCENT algorithm (5, 6). For the SCENT algorithm, abundance estimates were linked to the Entrez Gene ID using biomart and the protein-protein interaction network was obtained from Github as part of the SCENT algorithm. SCENT analysis included the 10000 most highly variable genes. Human embryonic stem cell and neural progenitor cell single cell sequencing data were obtained from gene expression omnibus (GSE75748 and GSE72056). Linear modeling was carried out in R using the lm package. Mixed-linear modeling was conducted using the R package lme4. For supplemental figure 3, centroids and inter-centroid distances were calculated using the clv package in R, bootstrapping analysis was performed with the boot package in R, and weighted linear modeling was performed using lm package in R.

Immunohistochemistry/Immunofluorescence: Human lung IPF tissue samples were fixed with 4% paraformaldehyde and paraffin-embedded (individual or combined in TMAs). Samples were

de-paraffinized and rehydrated 4 μm serial sections were stained with Hematoxylin-Eosin, or probed with the following antibodies: anti-SSEA4 (Biolegend, #330401, 1:50), anti-CD44 (Abcam, ab101531, 1:500), anti-MARCKS (Novus, NB110-58875SS, 1:500), anti-pro-collagen I (Abcam, ab64409, 1:500). For all antibodies, with the exception of anti-procollagen I, sections were subjected to antigen retrieval (BioCare, RV1000) for 20 min and Background SNIPER (BioCare, BS966) blocking reagent for 1h at RT. For anti-procollagen I, sections were treated with proteinase K (Millipore, 21627; working strength) for 5 min at RT. For IHC, prior to blocking, sections were treated with 3% hydrogen peroxide for 1 h, and after blocking, exposed overnight (4°C) to primary antibodies diluted in 10% Background SNIPER. For permanent staining, Novolink Polymer Detection Systems (Leica, RE7270-RE; manufacturer's recommendation) was used and developed with DAB chromogen (Covance, SIG-31042; manufacturer's recommendation). For anti-procollagen I, biotinylated anti-Rat (Vector Laboratories, #BA-4001) was used at 1:500 in 10% Background Sniper followed by Streptavidin-HRP (Covance, SIG-32254; working strength) and DAB chromogen. Slides were counterstained with hematoxylin and cover-slipped with Permount (Thermo Fisher Scientific, #SP15). For immunofluorescence, sections, after overnight primary antibody incubation sections were simultaneously stained with anti-Mouse Alexa Fluor 488 (Invitrogen, A11029, 1:1000) and anti-Rabbit Alexa Fluor 594 (Invitrogen, A11072, 1:1000) for 2 h at RT and cover-slipped with Prolong Gold Antifade reagent with DAPI (Thermo Fisher Scientific, P36931).

1. Larsson O, Diebold D, Fan D, Peterson M, Nho RS, Bitterman PB, Henke CA. Fibrotic Myofibroblasts Manifest Genome-Wide Derangements of Translational Control. In: Barnes PJ, editor. *PLoS ONE* 2008;3:e3220–12.
2. van der Maaten L, Hinton G. Visualizing Data using t-SNE. *Journal of Machine Learning Research* 2008;9:2579–2605.
3. van der Maaten L. Accelerating t-SNE using Tree-Based Algorithms. *Journal of Machine Learning Research* 2014;15:3221–3245.
4. Lin P, Troup M, Ho JWK. CIDR: Ultrafast and accurate clustering through imputation for single-cell RNA-seq data. *Genome Biology* 2017;18:59.
5. Enver T, Teschendorff AE. Single-cell entropy for accurate estimation of differentiation potency from a cell’s transcriptome. *Nature Communications* 2017;8:1–15.

6. Banerji CRS, Miranda-Saavedra D, Severini S, Widschwendter M, Enver T, Zhou JX, Teschendorff AE. Cellular network entropy as the energy potential in Waddington's differentiation landscape. *Nature Publishing Group* 2013;3:1129–7.
