# Supplementary material for: Single-cell RNA sequencing reveals that lung mesenchymal progenitor cells in IPF exhibit pathological features early in their differentiation trajectory
Source: Sci Rep. 2020 Jul 7;10:11162. doi: 10.1038/s41598-020-66630-5 (PMC7341888; doi:10.1038/s41598-020-66630-5)
Supplement: Supplementary file 4 — Supplementary Information 4. [file 41598_2020_66630_MOESM4_ESM.docx]

Single-cell RNA sequencing reveals that lung mesenchymal progenitor cells in IPF exhibit pathological features early in their differentiation trajectory

Daniel J. Beisang MD PhD^1^, Karen Smith PhD^2^, Libang Yang PhD^2^, Alexey Benyumov PhD^2^, Adam Gilbertsen^2^, Jeremy Herrera PhD^3^, Eric Lock PhD^4^, Emilian Racila MD^5^, Colleen Forster^6^, Brian J. Sandri PhD^7^, Craig A. Henke MD^2^, Peter B. Bitterman MD^2,†^

1. University of Minnesota, Department of Pediatrics, Division of Pediatric Pulmonology.
2. University of Minnesota, Department of Medicine, Division of Pulmonary, Allergy, Critical Care and Sleep Medicine.
3. University of Manchester, School of Biological Sciences, Division of Cell Matrix Biology & Regenerative Medicine.
4. University of Minnesota, School of Public Health, Division of Biostatistics.
5. University of Minnesota, Department of Laboratory Medicine and Pathology.
6. University of Minnesota, Clinical and Translational Science Institute.
7. University of Minnesota, Department of Pediatrics, Division of Neonatology.

† To whom correspondence should be addressed. Peter Bitterman, MD, Division of Pulmonary, Allergy, Critical Care and Sleep Medicine, 420 Delaware Street, SE, Minneapolis, MN 55455. Telephone 612-626-3773. Bitte001@umn.edu.
